# Prognostic factors and outcomes of early-stage small cell neuroendocrine carcinoma of the cervix: 37 cases from a single center

**DOI:** 10.7717/peerj.6868

**Published:** 2019-05-03

**Authors:** Dandan Zhang, Xiaoxin Ma

**Affiliations:** Department of Obstetrics and Gynecology, Shengjing Hospital Affiliated to China Medical University, Shengyang, China

**Keywords:** Neuroendocrine carcinoma, Prognosis, Small cell, Uterine cervix

## Abstract

**Background:**

The objective of this study is to investigate small cell neuroendocrine carcinoma of the cervix (SCCC), using a retrospective clinicopathological characteristic and treatment approach.

**Method:**

We retrospectively analyzed cases of early-stage SCCC, identified between 2006 and 2016, in women who received radical surgery and adjuvant chemotherapy with or without radiotherapy. Kaplan–Meier and one-way ANOVA analyses were performed.

**Result:**

A total of 37 cases of SCCC are presented in this study, of which 21 had stage IB1 SCCC, 12 had stage IB2, 3 had stage IIA1, and 1 had stage IIA2. All patients were treated with radical surgery and adjuvant chemotherapy, specifically, 26 with radical surgery followed by adjuvant chemotherapy plus radiation and 11 with neoadjuvant chemotherapy (NACT) followed by radical surgery. After a median follow-up time of 27 months (range, 8–115 months), the 2-year and 5-year disease-free survival rate for all patients was 51.9% and 34.1%, respectively, and the overall survival rate was 60.3% and 38.6%, respectively. Univariate analysis showed that International Federation of Gynecology and Obstetrics (FIGO) stage and tumor size may be a predictor of a poor prognosis. NACT and adjuvant radiation did not improve survival over adjuvant chemotherapy alone but should not be a significant independent prognostic factor for survival.

**Conclusion:**

Even in patients with early-stage SCCC, the prognosis is poor, although FIGO stage and tumor size may act as surrogate factors prognostic of survival.

## Introduction

Small-cell neuroendocrine carcinoma is a rare malignant tumor of the cervix (SCCC), accounting for 0.5–1% of all cervical cancer (Morris et al., 1992). It is characterized by early nodal and distant metastases, resulting in a poor prognosis (Miller et al., 1991).

The diagnosis of cervical neuroendocrine cancer is most commonly based upon the precise histopathological and immunohistochemical certification of neuroendocrine features. The first approach at unifying the terminology was in 1996 when the College of American Pathologists and the National Cancer Institute sponsored a workshop and proposed standardized terminology for neuroendocrine tumor of the cervix. The tumors are divided into four categories: typical carcinoid tumor, atypical carcinoid tumor, large-cell carcinoma, and small-cell carcinoma (SCC) (Albores-Saavedra et al., 1997).

Because of the rarity of the disease, most reported series have been small and from single institutions and prognostic factors, such as the tumor size, lymph-node metastases, stage, margin status, smoking, and age have been examined with mixed results (Xie et al., 2017; Zhou et al., 2016; Lee et al., 2015; Zivanovic et al., 2009). Early-stage carcinoma had a 5-year survival rate of 30–46%, while advanced-stage tumors had a low rate of survival at just 0–15% (Chan et al., 2003). Yet the prognosis for SCCC is much worse than that of squamous carcinoma and adenocarcinoma of the cervix and the patient with early-stage SCCC must face a depressing clinical outcome. The therapeutic approach for SCCC is a challenge for the gynaecologist and the therapeutic modalities are similar to small-cell lung cancer. The purpose of this study is to assemble the cases of SCCC, and to clarify the clinicopathologic features and prognosis for the disease.

## Materials and Methods

All of the surgically treated patients who were diagnosed with early-stage (IB1-IIA2) SCCC were identified from the Shengjing Hospital affiliated to China Medical University in China between January 2006 and December 2016 in this retrospective study. All patients involved in this study gave their informed consent. Institutional Review Board approval from the ethics committee of Shengjing Hospital affiliated to China Medical University was obtained for this study (2017ps037k). All histopathologic review was checked by two pathologists. The histological definition of SCCC is that the nuclear/cytoplasmic ratio is significantly high, the nucleus is hyperchromatic, the chromatin is fine, and the nucleoli are inconspicuous or rarely conspicuous, which is based on the International Classification of Disease for Oncology, third edition (ICD-O-3). Patients who did not meet the pathological diagnostic criteria, refused post-operation adjuvant treatment, or were lost to follow-up were excluded.

All patient and clinicopathological characteristics were extracted from the electronic medical records including age, tumor size, International Federation of Gynecology and Obstetrics (Pecorelli & FIGO Committee on Gynecologic Oncology, 2009) stage, tumor homology (simple/mixed SCCC), lymph node metastases, depth of stromal invasion (DSI), lymphovascular space invasion (LVSI), surgical margin involvement, parametrial positive, and preoperative/postoperative adjuvant therapy method.

All patients underwent a radical hysterectomy (RH) and were counselled to undergo chemotherapy, drawing on the data for treating small-cell lung cancer. Preoperative adjuvant treatment for patients who had a tumor ≥4 cm and a postoperative two-drug chemotherapy regimen was cis-platinum(C)/carboplatin(P) with etoposide(E) or cis-platinum(C)/carboplatin(P) with paclitaxel(T) for three to six cycles. Cisplatinum is typically dosed at 60 mg/m2 IV on day 1 and etoposide at 120 mg/m2 IV on days 1–3, or carboplatin AUC = 5 and etoposide every 21 days in etoposide region, which was recommended in the society of gynecologic oncology (SGO) in 2011 and the gynecologic cancer InterGroup (GCIG) in 2014. In paclitaxel region, paclitaxel 175mg /m2 and cisplatin 60 mg/m2 IV, or paclitaxel and carboplatin AUC = 5 were given every 21 days. Adjuvant radiotherapy after chemotherapy was suggested to each patient who experienced a recurrence in more than one region.

After radical surgery, patients were seen regularly every 3 months to monitor for disease recurrence, which included a routine review of symptoms and a pelvic exam. Chest X-ray, pelvic ultrasound, and periodic imaging such as CT or PET-CT, were used in the interim for early detection for survival prognosis.

Cancer-related death was selected as the primary endpoint, and the secondary endpoint was tumor recurrence. Disease-free survival (DFS) was calculated as the date of RH operation to cancer recurrence or as reviewed in the final follow-up. Overall survival (OS) was calculated from the date of RH to death or as reviewed in the final follow-up.

The Kaplan–Meier method and log-rank test were used to evaluate OS and DFS. The one-way ANOVA analysis was used to estimate the independent prognostic factors for OS and DFS. All data was analyzed using SPSS 23.0 software (SPSS, Chicago, IL, USA). Statistical differences were considered when *p* < 0.05.

## Results

Between January 2006 and December 2016, data from a total of 42 SCCC patients was processed at our institution. Two patients were excluded because they did not have SCC in the histopathologic review, and three were excluded because systemic adjuvant chemotherapy was not performed. A total of 37 patients were included in the study.

This study was approved by the Regional Ethics Committee of our hospital and all patients signed informed consents. The median age of the patients was 38 years (range, 22–61 years). Among these patients, there were 21 cases of FIGO stage IB1 (56.76%), which was the most common stage, 12 cases of stage IB2 (32.43%), three cases of stage IIA1 (8.11%), and one case of stage IIA2 (2.70%).

Among the 37 cases reviewed, 28 cases were confirmed on review for simple SCC in pathologic diagnosis, and nine cases with admixed SCCC carcinoma. Three cases were large-cell carcinoma with SCCC, two cases were seen as atypical carcinoid carcinoma with SCCC, one case was typical carcinoid carcinoma with SCCC, two cases were diagnosed as squamous carcinoma with SCCC, and one case was adenocarcinoma with SCCC.

Among these patients, 23 accepted a preoperative human papillomavirus (HPV) test; 17 of them were HPV 18–positive, four cases were HPV 16–positive and one case was infected with both, another one was infected with other types of HPV. The clinical and pathological characteristics are summarized in Table 1.

**Table 1:** Patient clinical and pathological characteristics.

Variables	Cases (%)
Age	
<40 years	20 (54.05)
≥40 years	17 (45.95)
HPV infection	
HPV 18+	17 (77.27)
HPV 16+	4 (18.18)
HPV 16+ 18+	1 (4.54)
Tumor homology	
Smiple SCC	28 (75.68)
Mixed SCC	9 (24.32)
FIGO stage	
IB1	21 (56.76)
IB2	12 (32.43)
IIA1	3 (8.10)
IIA2	1 (2.70)
Lymphovascular infiltration	23 (62.16)
Lymph node metastasis	19 (51.35)
Deep stromal infiltration	27 (72.97)
Parametrial infiltration	0 (0)
Vaginal margin infiltration	0 (0)
Postoperative chemotherapy	
Cisplatin/Carboplatin+Etoposide	26 (70.27)
Cisplatin/Carboplatin+Paclitaxel	11 (29.73)
Postoperative radiotherapy	15 (40.54)
Preoperative chemotherapy	13 (35.14)

A total of 26 patients (70.27%) in the registry received a two-drug regimen, which was comprised of cisplatin/carboplatin and etoposide as the primary therapy after surgery, whereas 11 (29.73%) patients received cisplatin/carboplatin and paclitaxel. Radical surgery was followed by adjuvant radiation in 15 patients (48.20%) who developed recurrence and metastasis, and neoadjuvant chemotherapy (NACT) was followed by radical surgery in 13 cases (35.14%) for local tumor size over four cm. Four patients with more than two regions affected accepted radiotherapy and chemotherapy with the original regimen. One patient rejected radiotherapy for recurrence treatment, and lived for 6 months with simple chemotherapy.

**Figure 1:**
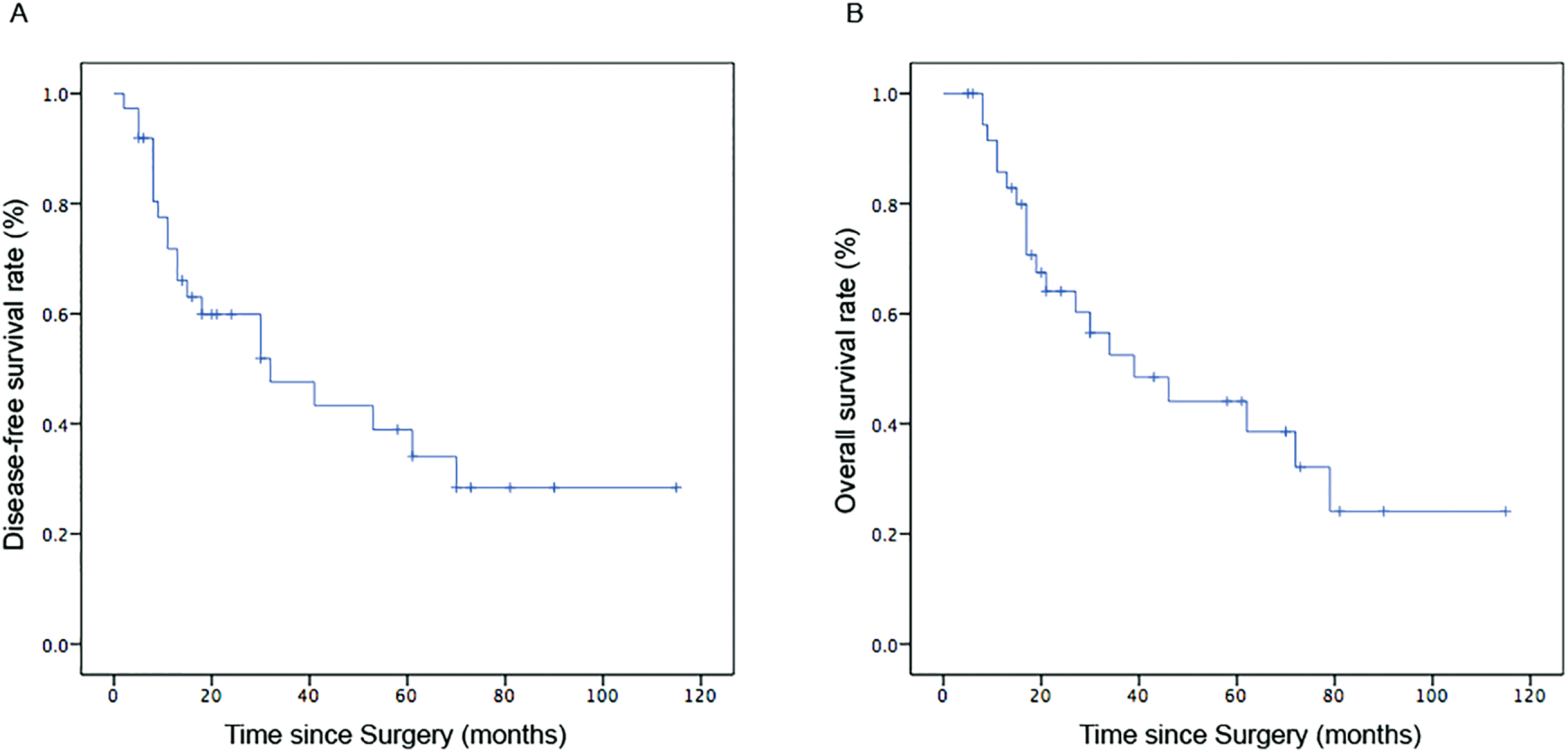
Survival curves of all 37 patients with early-stage SCCC. (A) DFS and (B) OS.

A follow-up after 8–115 months (median 27 months) was completed for these 37 patients; the median DFS was 32 months, the median OS time was 39 months, simultaneously. Among 37 SCCC patients with FIGO stage IB-IIA, the 2-year and 5-year DFS rates of all patients were estimated as 51.9% and 34.1%, while 2-year and 5-year OS rates were 60.3% and 38.6% (Fig. 1). Patients with early stage carcinoma had better DFS and OS (*p* < 0.05), while those with small tumor size had better DFS (*p* < 0.05). No differences in DFS and OS were observed, based on age, tumor homology, lymph node involvement, DSI, LVSI, and postoperative/preoperative adjuvant treatment modalities.

**Figure 2:**
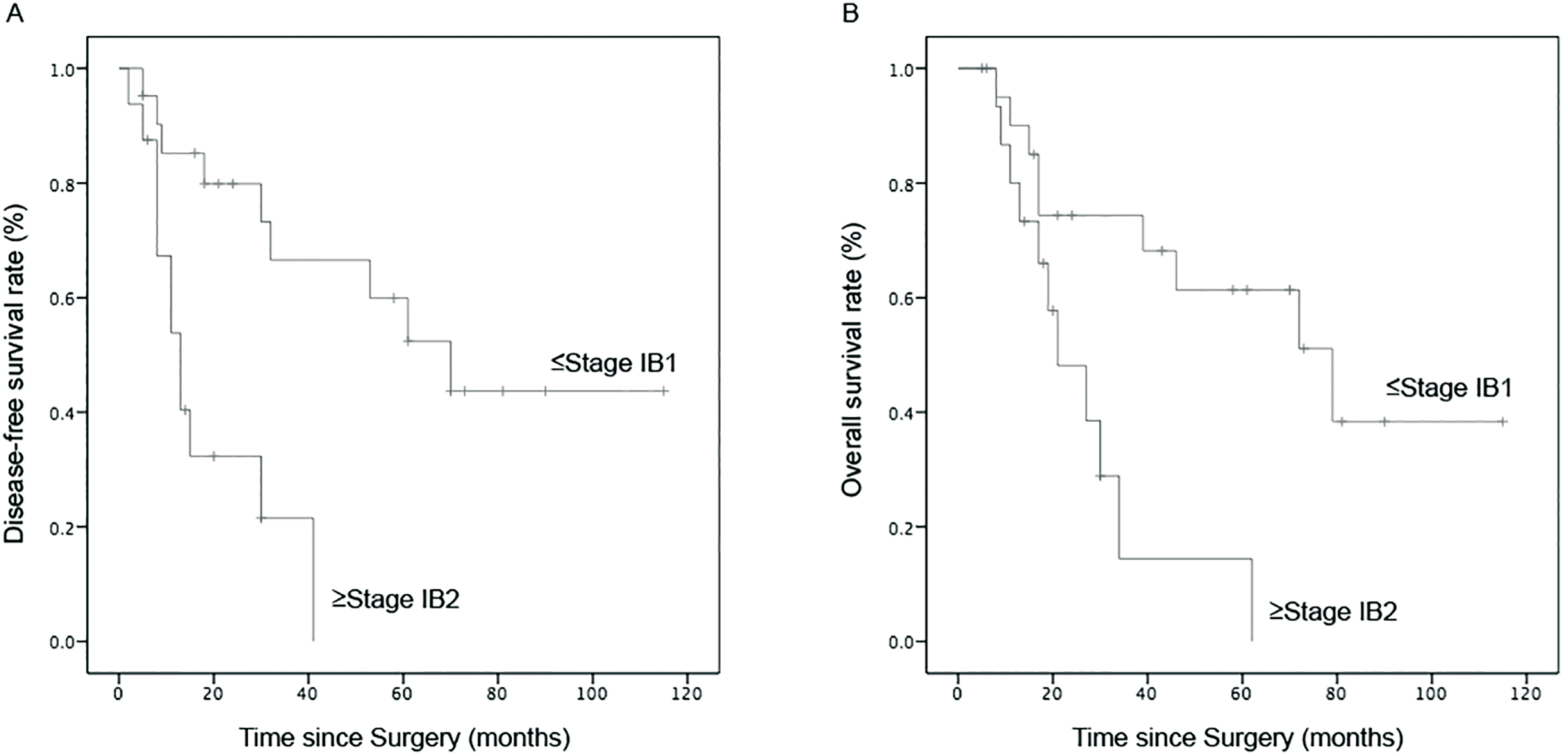
Comparison of survival curves in patients with different stages of SCCC. (A) DFS and (B) OS.

**Figure 3:**
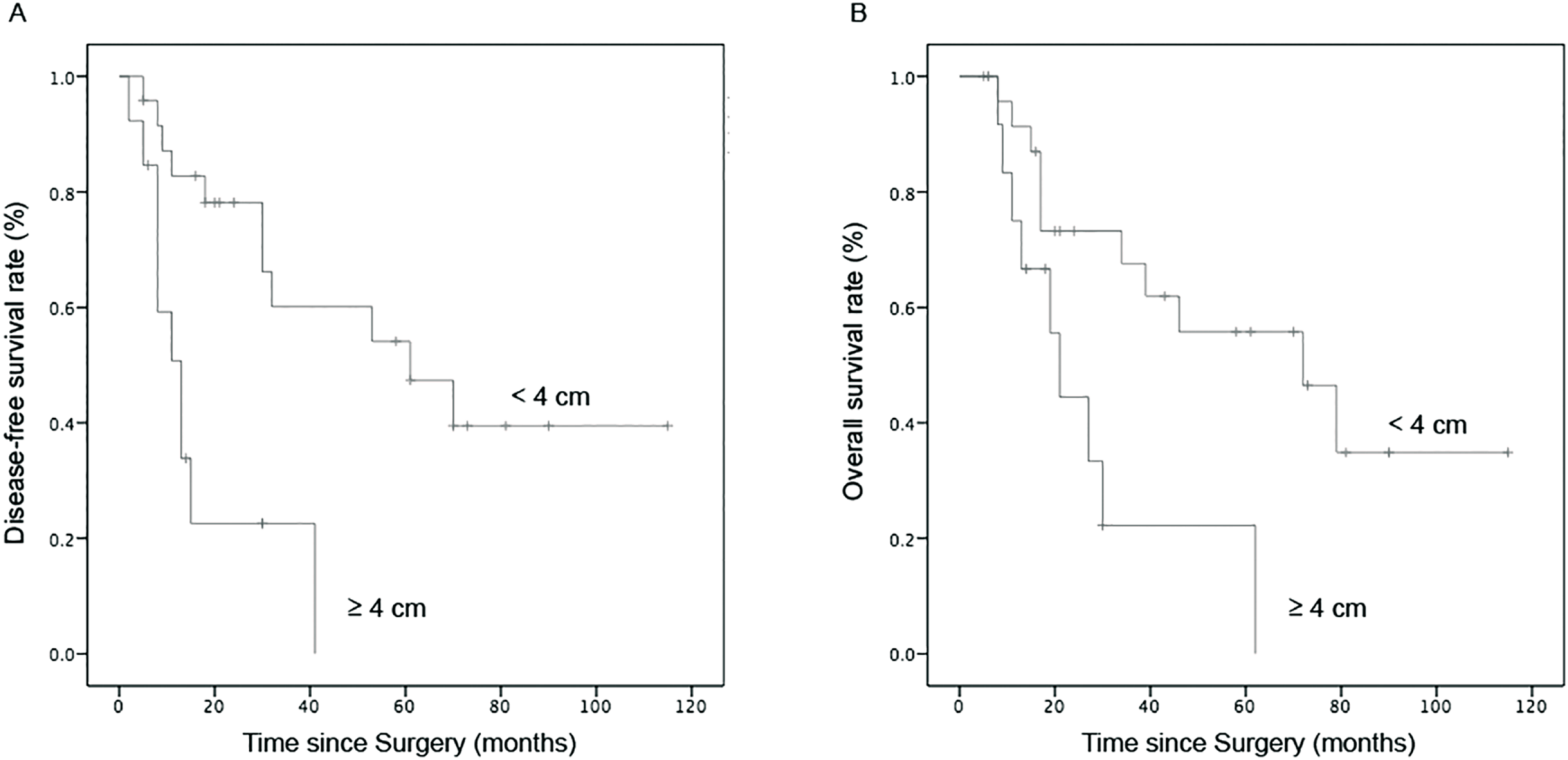
Comparison of survival curves in patients with different tumor sizes of SCCC. (A) DFS and (B) OS.

We analyzed multiple clinicopathologic features for potential prognostic survival values of SCCC patients (Table 2). Univariate analysis showed that the 2-year DFS of patients with FIGO stage ≤IB1 (*p* = 0.01) and tumor size <4 cm (*p* = 0.01) was significantly prolonged, whereas, the 2-year OS was just prolonged in patients at stage ≤IB1 (*p* = 0.04; Fig. 2). The 2-year OS of patients whose tumor size was <4 cm was 67.6%, compared with 33.3% for those with tumor size ≥4 cm, and the median survival time at 72 months was also longer than 21 months for patients whose tumor size was ≥4 cm (*p* = 0.07) (Fig. 3). Although the data was not statistically significant, there was a tendency toward prolonged OS in patients with negative LVSI (*p* = 0.231) and DSI <1/2 (*p* = 0.153). In contrast, tumor histological homogeneity (*p* = 0.452) and pelvic/para-aortic lymph node metastasis (LNM) (*p* = 0.874) were not prognostic factors for survival.

**Table 2:** Univariate analysis of 2-year DFS and OS based on pathological factors in early-stage SCCC.

	Cases	2-years DFS (%)	*p*-value	2-years OS (%)	*p*-value
Age			0.659		0.742
<40 years	20	62.3		64.8	
≥40 years	17	51.5		56.4	
Tumor homology			0.452		0.812
Smiple SCC	28	54.3		56.9	
Mixed SCC	9	59.0		63.7	
FIGO stage			0.01		0.04
≤IB1	21	73.2		78.2	
≥IB2	16	21.5		38.5	
Tumor size (cm)			0.01		0.07
<4	24	66.1		67.6	
≥4	13	0		33.3	
Pelvic/para-aortic LNM			0.874		0.812
Yes	19	58.9		63.8	
no	18	52.6		54.2	
LVSI			0.158		0.231
Yes	23	49.5		51.4	
no	14	72.4		75.5	
DSI			0.095		0.153
Yes	27	46.8		49.1	
no	10	68.2		70.2	
Preoperative chemotherapy			0.064		0.162
Yes	11	32.8		36.5	
no	16	72.1		73.4	
Postoperative chemotherapy			0.815		0.856
Cisplatin/Carboplatin+Etoposide	26	55.2		55.9	
Cisplatin/Carboplatin+Paclitaxel	11	59.2		64.2	
Postoperative radiotherapy			0.158		0.174
Yes	15	48.2		49.1	
no	22	67.3		70.3	

A total of 16 patients developed recurrence and metastasis: three in the pelvic region, four in the liver, three in the lung, four in the adrenal glands, three in the bone, two in the brain, and two in the para-aortic lymph nodes. Most of the patients received adjuvant radiotherapy after chemotherapy with recurrence in one or two regions, and four patients with invasion in more than two regions accepted radiotherapy and chemotherapy with the original regimen. Only one patient rejected radiotherapy for recurrence treatment and lived for 6 months with simple chemotherapy. Of the 16 patients, 14 patients died, and the remaining two patients lived with tumor recurrence.

We also assessed the influence of treatment modalities on survival. Survival dates were analyzed with respect only to the treatment modality. Patients who received an etoposide combination of chemotherapy had a 2-year DFS of 55.2% compared to 59.2% in those on a regimen of a paclitaxel combination (*p* = 0.815). Furthermore, patients with early-stage disease who underwent adjuvant radiotherapy had a 2-year DFS of 48.2% compared to 67.3% in those without radiotherapy (*p* = 0.158). The univariate analysis did not identify postoperative chemotherapy in different regimens (*p* = 0.856) or adjuvant radiotherapy (*p* = 0.174) as prognostic factors for 2-year OS in the entire cohort. Due to the limited number of patients in this study, patients who received adjuvant radiotherapy and different chemotherapy regimens could not be demonstrated to have a significant survival advantage.

## Discussion

Small cell neuroendocrine carcinoma of the cervix is a rarely encountered cervical cancer. Epidemiological studies have shown that SCCC accounts for an incidence of only 1–3% of cervical cancer cases (Tsunoda et al., 2005; Crowder & Tuller 2007); However, the high degree of invasiveness and early distant metastasis leads to a poor prognosis at the time of the diagnosis of SCCC (Chan et al., 2003; Viswanathan et al., 2004). Early SCCC may have a 5-year survival rate between 30% and 46%, while the advanced stages have a poor 5-year survival rate as low as 1–15% (Nasu et al., 2011). In our study, all patients who were diagnosed with stage I of IB1 through colposcopy biopsy or Loop Electrosurgical Excision Procedure showed difficulties in early diagnosis to some extent.

International Federation of Gynecology and Obstetrics stage, large tumor size, LNM, deep cervical stromal invasion, smoking, and pure small-cell histology are always suggested as indicators of a poor prognosis in SCCC (Xie et al., 2017; Zhou et al., 2016). However, previous studies were limited by sample sizes and enrolled patients at all stages, which was interrelated with rare incidence (Gersell et al., 1988; Cohen et al., 2010). In fact, the early-stage and advanced-stage patients might be intrinsically different, not only in prognostic factors, but also in treatment strategies. In our study, we only focused on patients with early-stage SCCC and conducted univariate analysis for the limited sample size. Our research revealed that only stage and tumor size were risk factors for DFS, and stage was a high-risk factor for OS alone. The survival outcomes for our study, which is a simple center report, are consistent with others. The 5-year OS of our surgically treated patients with stages IB1-IIA2 was 38.6%, below the 5-year OS of 50% in Chen et al.’s (2015) report for IB-IIA patients in the largest Taiwanese study, and 46.6% reported by Lee et al. (2007) who conducted a multi-center study in Korea. Other factors require further consideration. First, an increasing number of studies have shown that patients undergoing laparoscopic surgery have a higher incidence of postoperative recurrence than those undergoing conventional surgery; secondly, Chen et al. (2015) believed that smoking was a poor prognostic factor for cervical cancer and SCCC. In our case, there are not enough records about female patients’ smoking to accurately analyze this factor.

The biological behavior of SCCC is different than conventional cervical cancers such as squamous cell carcinoma or adenocarcinoma. SCCC generally does not invade the surface epithelium of the cervix but diffuses into the interstitial tissue. In our study it was true that 62.16% of all cases had vascular invasion and 51.35% had LNM at stage IB1–IIA2. The prognostic value of the SCCC lymph node status is still controversial. Xie et al. (2017) and Chan et al. (2003) found that lymph node status was an independent prognostic factor for SCCC patients’ survival. Chen et al. (2015) found that patients with detected histological LNM showed worse 5-year OS than negative node patients. On the other hand, other studies have shown that lymph node status was not associated with the SCCC prognosis (Lee et al., 2015; Zivanovic et al., 2009; Kuji et al., 2013). In our study, we analyzed the data of 37 patients, of whom 51.35% had nodal metastases. That was higher than the number of positive lymph nodes found in conventional cervical cancer (squamous cell carcinoma or adenocarcinoma) at the same stage, but not significantly related to prognosis. Intaraphet et al. (2014) reported that lymph node status had no relationship with prognosis in early-stage patients receiving surgery only but was a high-risk factor for recurrence in all patients. Wang et al. (2012) and Lee et al. (2015) also used the statistics of surgery-only patients for analyzing lymph node status and prognosis, and the result was irrelevant. Castelnau-Marchand et al. (2018) reported that lymph node involvement at the time of diagnosis was significant for OS in locally advanced neuroendocrine cervical carcinoma. Furthermore, the rarity of the disease limits the number of patients, which also affects the prognostic results. This result indicated that the lymph node status in SCCC patients deserves further study.

Small cell neuroendocrine carcinoma of the cervix is like the small-cell cancer arising from the pulmonary bronchi and was first described by Reagan, Hamonic & Wentz (1957). Thus, some reports suggest that chemotherapeutic regimens should be similar to those used in the treatment of lung small-cell neuroendocrine carcinoma. Because these tumors share pathological and behavioral similarities, aggressive treatment regimens from clinical trials for small cell neuroendocrine carcinoma of the lung may be appropriate for the treatment of similar tumors for cervical cancer. However, SCCC appears to be related to HPV, the regimen of platinum-based chemotherapy with Paclitaxel is also considered. SCCC has been reported to be associated with HPV 16 and HPV 18 (Conner et al., 2002; Abeler & Kjørstad 1991). Some studies suggested that HPV 18 is a viral type specifically associated with SCCC. Ishida et al. (2004) reported that in total, 31 SCCC cases (65%) were HPV 18 positive, and Masumoto et al. (2003) found 8 HPV 18 positive patients in 10 SCCC cases. Our data also showed that of the SCCC cases that received a preoperative HPV DNA test, the majority were associated with HPV 18. While examining postoperative adjuvant therapy, Chang et al. (1998) compared the first group which used cyclophosphamide, doxorubicin, and vincristine (CAV), similar to treatment for small-cell lung cancer therapy, and a second group using other chemotherapy achieved a significantly higher survival rate. Our data compared a group given an etoposide-combination and a group given a paclitaxel-combination and found no differences in either DFS (*p* = 0.815) or OS (*p* = 0.815).

In our study, surgery with preoperative chemotherapy was mostly applied in patients with local advanced-stage (tumor size ≥4 cm) cancer. For early-stage but bulky disease (≥4 cm), the SGO and GCIG guidelines propose NACT, followed by surgery in appropriate responders, and then further systemic chemotherapy with or without radiation (Satoh et al., 2014; Gardner, Reidylagunes & Gehrig, 2011). The cause of the poor survival rates of patients receiving NACT pre-operation is not clear, however, it can be assumed that patients with tumor size over four cm, receiving NACT, may have more high-risk factors of recurrence such as parametrial invasion, LVSI, and DSI. On the other hand, patients who reaccepted adjuvant radiotherapy also exhibited poor survival in our study. Lee et al. (2015) reported that the 5-year OS rate of the early-stage patients who received adjuvant radiotherapy plus chemotherapy was 40.2%, but 53.9% in adjuvant-only chemotherapy patients. A large amount of clinical data shows that adjuvant radiation decreases pelvic recurrence, but does not improve OS because of the inability to prevent distant metastases. Optimal management remains ill-defined but an intensive treatment with chemoradiation followed by a brachytherapy boost could allow most patients to benefit from surgery, especially for locally advanced neuroendocrine cervical carcinoma in the Castelnqu-Marchand result (Castelnau-Marchand et al., 2018). Only two patients developed pelvic recurrence compared with distant metastases in our research, so it is likely that adjuvant chemotherapy may enhance survival relative to radiation, due to the higher incidence of distant metastases in early-stage SCCC.

In brief, SCCC remains an anagogic and elusive disease to study due to its rarity. Many gynecologists expect to see a solution to the disease not only in the laboratory but also in clinical trials. An online database for this disease is needed for performing clinical trials in the patient population and proposing a regimen of treatment. Currently, molecular testing and using targeted agents from tumor samples to treat mutations will provide a new concept for tumor gene mutation and repair those with targeted drugs.

## Supplemental Information

10.7717/peerj.6868/supp-1 Supplemental Information 1 Dataset S1: Raw data of overall survival and progression free survival.

## Additional Information and Declarations

### Data Availability

The following information was supplied regarding data availability: The raw measurements are available as a Supplementary File.
